# Chloroplast transformation for bioencapsulation and oral delivery using the immunoglobulin G fragment crystallizable (Fc) domain

**DOI:** 10.21203/rs.3.rs-3073879/v1

**Published:** 2023-07-26

**Authors:** Lisa LaManna, Chih-Hsuan Chou, Hanqin Lei, Elisabeth R. Barton, Pal Maliga

**Affiliations:** 1 Waksman Institute of Microbiology, Rutgers University, Piscataway, NJ 08854, USA.; 2 Department of Plant Biology, Rutgers University, New Brunswick, NJ 08901, USA.; 3 Department of Applied Physiology & Kinesiology, University of Florida, College of Health and Human Performance, Gainesville, FL, 32611, USA

## Abstract

Proinsulin Like Growth Factor (prolGF1) and myostatin (Mstn) regulate muscle regeneration when intravenously delivered. We set out to test if chloroplast bioencapsulated forms of these proteins may serve as a non-invasive means of drug delivery through the digestive system. We created tobacco (*Nicotiana tabacum*) plants carrying *GFP-Fc1, proIGF-I-Fc1,* and *Mstn-Fc1* fusion genes, in which fusion with the immunoglobulin G Fc domain improved both protein stability and absorption in the small intestine. No transplastomic plants were obtained with the *Mstn-Fc1* gene, suggesting that the protein is toxic to plant cells. proIGF-I-Fc1 protein levels were too law to enable *in vivo* testing. However, GFP-Fc1 accumulated at a high level, enabling evaluation of chloroplast-made Fc fusion proteins for oral delivery. Tobacco leaves were lyophilized for testing in a mouse system. We report that the orally administered GFP-Fc fusion protein (5.45 μg/g GFP-Fc) has been taken up by the intestinal epithelium cells, evidenced by confocal microscopy. GFP-Fc subsequently entered the circulation where it was detected by ELISA. Data reported here confirm that chloroplast expression and oral administration of lyophilized leaves is a potential delivery system of therapeutic proteins fused with Fc, with the advantage that the proteins may be stored at room temperature.

Preservation of muscle mass and countering its loss is vitally important goal in many clinical situations, including disuse atrophy, aging-related loss of muscle (sarcopenia), neuromuscular disease, and muscle wasting (cachexia). Two key regulatory agents are insulin-like growth factor I (IGF-I) and myostatin (Mstn), which represent the “yin and yang” of muscle mass modulation ^1^. IGF-I is an anabolic hormone produced in the liver, skeletal muscles, bones, and brain, that is known to stimulate proliferation and differentiation of many cell types and plays an important role in tissue renewal and repair ^2^. Myostatin, a member of the transforming growth factor-β (TGF-β) superfamily, is a powerful negative regulator of skeletal muscle mass in mammalian species ^3,4^. Nearly all clinical studies or FDA approved treatments for muscular diseases regarding these agents are dependent on needle-based application, the efficacy of which is questionable, particularly as the administration of low levels of growth factors and the limitations of the circulatory system to distribute proteins to muscle tissues are recognized constrains to the benefits of systemic delivery of recombinant proteins like IGF-I ^5–8^. In addition, high cost of fermentation facilities, prohibitively expensive protein purification technologies, cold storage/ transportation and sterile delivery make protein drugs unaffordable for a large global population earning < $2/day ^9,10^. Expression and bioencapsulation of recombinant proteins for muscle diseases using chloroplast transformation biotechnology can procure orally available therapies to replace injections. Furthermore, this efficient system has economic potential by obviating costly purification procedures. This may, in turn, increase accessibility of muscle atrophy treatments to a wider population.

The highly conserved *Igf1* gene encodes a prepropeptide consisting of a signal peptide to direct secretion, the mature IGF-I peptide, and a C-terminal extension called the E-peptide ^11,12^. The predominant *Igf1* isoform, *Igf1a,* constitutes 90–95% of the mammalian *Igf1* mRNA transcripts ^13,14^. Retention of the E-peptide (proIGF-I) has been shown to enhance receptor binding *in vitro* compared to the mature IGF-I alone ^15^. Further, removal of the initial 3 residues of the mature protein (GPE) increases IGF-IR activity, in part through a reduction of affinity for the family of IGF binding proteins ^16^. Thus, we set out to express the pro form of IGF-I with or without GPE, and without the signal peptide.

Myostatin (Mstn) encodes a prepropeptide consisting of a signal peptide, latency-associated peptide (LAP) and mature growth factor (GF) protein. The LAP, or propeptide, is important for myostatin maturation and proper folding ^17–19^, but also remains associated with the mature protein after secretion into the extracellular matrix via strong noncovalent bonds that impedes GF activity. Additional processing of the propeptide by a member of the BMP1/Tolloid (TLD) family of metalloproteases destabilizes the propeptide, which results in the full activation of the myostatin GF. A single amino acid substitution in this processing site abolishes recognition by BMP1/TLD-like proteinases thereby preventing myostatin activity, which results in increased muscle mass when injected into mice ^20,21^. We sought to express recombinant myostatin propeptide containing a D76A mutation for BMP1 cleavage prevention, thus generating a dominant negative gene product which, upon uptake into the blood stream, is capable of binding mature GF and inhibiting the Smad2/3 signaling cascade that leads to muscle atrophy.

For efficient uptake by intestinal epithelial cells into the bloodstream it is necessary to fuse the chloroplast bioencapsulated proteins to a specific compound. Two systems are currently available and utilize N-terminal fusions to Cholera non-toxic B subunit and the protein transduction domain (PTD) for the expression of recombinant pro-IGF-I which has been codon optimized for expression in lettuce chloroplasts ^10,22^. Murine oral gavage trials with these fusions have demonstrated successful uptake into the bloodstream, accumulation in skeletal muscles of treated mice, and improvements if bone fracture healing in diabetic mice. While promising, the N-terminal fusions can impair effective receptor activation if the fusion peptide or remnants of non-IGF-I sequences remain following cleavage. This is because the N-terminus of IGF-I is important for receptor engagement ^23^. To avoid this issue, we look to generate an additional system for oral delivery of therapeutic proteins using the Immunoglobulin G (IgG) fragment crystallizable (Fc) domain for fusion of recombinant protein at the C-terminus, and a chloroplast expression system that consistently delivers high expression levels ^24–27^. We generated chloroplast transformation vectors carrying recombinant proIGF-I and Mstn propeptide fused to the IgG fragment crystallizable (Fc) domain to promote absorption through the epithelial lining of the small intestine ^28–30^. Transplastomic tobacco lines were generated, and heterologous protein expression was quantified and characterized in each of the generated transplastomic lines. We lyophilized the leaf tissues to concentrate the recombinant protein, then used the lyophilized tissue in murine oral-gavage trials and *in vitro* activity assays. Only GFP-Fc was expressed at sufficiently high levels to enable testing the orally delivered fusion protein. Our findings suggest chloroplast-bioencapsulated therapeutic drug delivery after fusion with the immunoglobulin G Fc domain is a compelling alternative to current strategies.

## Results

### Vectors for expression of the Fc fusion proteins in chloroplasts.

**Figure 1.**
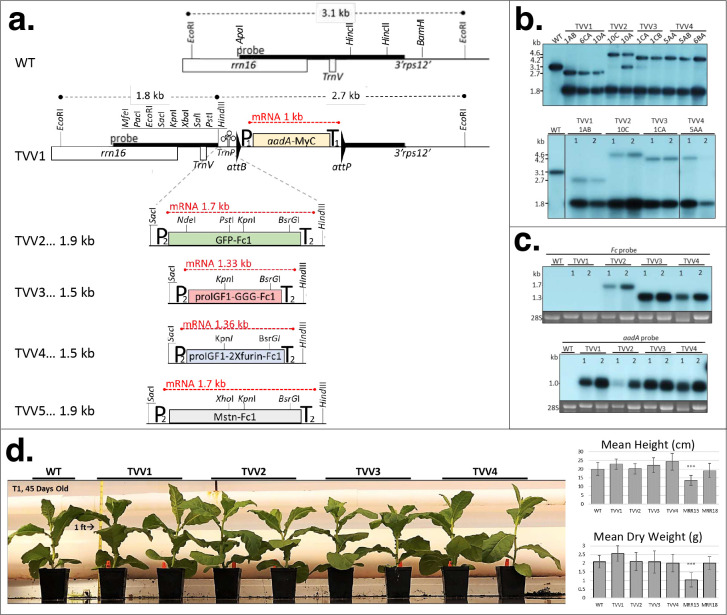
Tobacco Vaccine Vectors (TVV) for plastid transformation. (**a**) Map of the plastid targeting region of vectors TVV1 and TVV2. Plastid genes *rrn16*, *trnV* and the promoter region of *3’rps12* gene is shown. For further explanation see Results. (**b**) DNA gel blot confirms transgene integration into the chloroplast genome and the absence of non-transformed ptDNA copies. Total cellular DNA was digested with the *Eco*RI restriction endonuclease and probed with the 1.9-kb *Apa*I-*Bam*HI fragment (Fig 1a). Two μg of total cellular DNA was loaded per lane in 1% agarose gel. Wild-type ptDNA yields a 3.1 kb hybridizing fragment, and transplastomic TVV plants yield 2.7 and 1.8 kb fragments for TVV1, 4.6 and 1.8 fragments for TVV2, and1.8 and 4.2 kb fragments for TVV3 and TVV4. Blots prepared with DNA from T0 plants (upper panel) and T1 plants (lower) are shown. (**c**) TVV transcripts detected on RNA gel blots with Fc1 and *aadA* probe. The *GFP-Fc1* transgene yields a 1.7 kb transcript, *proIGF-I-Fc1NC* a 1.3 kb transcript, *proIGF-I-Fc1C* a 1.4 kb fragment, while the *aadA-cMyc* produces a 1 kb transcript. 2 μg of total cellular RNA was loaded per lane in 1.5% formaldehyde agarose gel. (**d**) Phenotype and biomass of TVV tobacco plants. Phenotypes are shown 45 days after germination. Plant height and dry weight were determined after approximately 54 days after germination. Above-ground plant dry weight was measured after drying in paper bags at 80°C for 5 days. Statistical analysis of three trials was performed with SAS using the Dunnett’s test to compare all lines to wild type (WT) after blocking. *P< 0.05, **P < 0.01, ***P < 0.001. Measurements are based on 56 plants, in three experiments. MRR15 and MRR18 plants were tested only twice.

The Tobacco Vaccine Vectors (TVV) used in the study are depicted in Fig. 1. The basic vector TVV1 (Fig. 1a) carries a spectinomycin resistance (*aadA*) marker gene in a *rbcL* plastid gene promoter/terminator cassette (P1/T1). The *aadA* coding region is translationally fused with cMyc tag to facilitate quantification of aminoglicosyde-3”-adenylyl transferase (AAD). The marker gene is flanked by minimal *attB* (34 bp) /*attP* (39 bp) sequences, which are target sites for the PhiC31 phage-site-specific integrase for post-transformation excision of the marker gene ^31^. Between the multiple cloning site and *attB* site we have included a tRNA (*trnP*), known to be efficiently processed in polycistronic mRNAs ^32,33^.

In vector pTVV2 we expressed a green fluorescent protein (GFP) fused with Fc at the C-terminus via 3x glycine linker (GFP-Fc1). Expression of proIGF-I fusion proteins contained either a noncleavable 3xglycine linker combined with deletion of GPE in TVV3 (proIGF-I-Fc1NC), or a cleavable proIGF-I-Fc1 fusion protein with a 2x furin linker and retention of GPE (proIGF-I-Fc1C) in TVV4 (Fig. 1a). Finally, the sequence for murine myostatin propeptide harboring a D76A mutation with Fc peptide at its C-terminus was expressed in TVV5. Each sequence was codon optimized for expression in chloroplasts using the OPTIMIZER program ^34^. The synthetic coding region was inserted in the P*rrn*La*tpH*/ T*his*/*thr* cassette. P*rrn*L*atpH* is a promoter-leader cassette, obtained by fusing the tobacco plastid rRNA operon promoter with the maize *atpH* gene leader. The maize *atpH* leader, in a dicistronic operon, yielded very high levels of GFP (23.5% of Total Soluble Protein or TSP) in potato leaves (pAI3) ^35^. We expected therefore, that the ribosomal RNA operon promoter (*Prrn*) fused with the 5’-UTR of the *Zea maize atpH* gene leader harboring a PPR10 RNA binding site should elicit a strong translation initiation signal. The cassette also contains a histidine-threonine attenuator (T*his*/*thr*) to stabilize the 3’ UTR of the mRNA ^36^. Each *Fc1* fusion transgene was inserted into the multiple cloning site of pTVV1, placing it upstream of the selectable spectinomycin resistance gene (*aadA*).

### Characterization of the transplastomic plants.

Sterile tobacco leaves were bombarded with TVV1, TVV2, TVV3, and TVV4 vector DNA. After bombardment the leaves were dissected into 1 cm^2^ sections and transferred to RMOP shoot regeneration medium containing 500 mg/L spectinomcyin, where wild type leaf sections bleach out and proliferate very slowly. Tentative transplastomic clones were identified as regenerating green shoots. The green shoots may be spontaneous spectinomycin resistant mutants, or plastid transgenic (transplastomic) events. Spontaneous mutants are resistant to spectinomycin only, while transplastomic events are resistant to both, spectinomycin and streptomycin, the property conferred by the *aadA* gene ^37^. Double resistance was confirmed in over 70% of the evaluated spectinomycin resistant clones for TVV1, TVV2, TVV3, and TVV4. The clones were obtained in 3 bombarded samples, a relatively high plastid transformation efficiency (Table 1).

To achieve uniformity of transformed plastid genomes, shoots were repeatedly regenerated from the leaves of transplastomic plants. The clones are designated by the construct name and serial number. For example, TVV1-3AD is a plant that was obtained by transformation with vector TVV1, and was designated event No. 3. Because the plants regenerated from the initial event often contain mixed wild-type and transformed plastid genome copies, it is routine to regenerate new shoots from the leaves of primary events expecting that new shoots develop from a small cluster of cells which contain only transformed ptDNA copies. Shoots regenerated from the same leaf section are identified by the letters of the alphabet. The addition of AD indicates that the plant TVV1-3AD went through two cycles of consecutive shoot regeneration (Fig.1b).

Chloroplast transformation was confirmed by DNA gel blot analyses in multiple, independent clones by probing *Eco*RI-digested total cellular DNA. Hybridization with the 1.9-kb *rrn16* probe yielded a 3.1 kb hybridization band for the wild type ptDNA, a 2.7 kb band for TVV1, a 4.6 kb band for TVV2 and 4.2 kb bands for TVV3 and TVV4. The *Eco*RI cleavage site within the multiple cloning site yielded an additional 1.8 kb hybridization band in each transplastomic plant lines. The 3.1 kb band in TVV2-10A plants indicates that wild-type ptDNA is still present indicating that the plant is still heteroplastomic (Fig. 1b, upper panel). However, the wild-type fragments segregated away in the T_1_ seed generation. Individuals from a single line, indicated in the T_1_ DNA gel blot analysis, were used to carry out the remaining experiments (Fig. 1b, lower).

The predicted transplastomic fragment sizes for TVV5 were 1.8 kb and 4.1 kb. We obtained a relatively small number of spectinomycin resistant clones (Table 1), but never obtain events with the predicted fragment sizes. We concluded therefore, that the myostatin propeptide-Fc fusion protein is toxic to plant calls and high-level expression is incompatible with the cellular metabolism due to potential interference with the plant brassinosteroid metabolism ^38^.

Northern blot analysis was performed to visualize TVV transcripts in the transplastomic lines (Fig. 1c). Total leaf RNA was extracted, separated in agarose gels, transferred to nitrocellulose membranes and then hybridized with the Fc probe and the *aadA* probe. The transcript size detected by the Fc probe is approximately 1.7 kb in TVV2 and 1.3 kb in TVV3 and TVV4 (Fig. 1c). The absence of hybridizing band larger than 1 kb indicates the absence of readthrough transcripts from the Fc fusion genes due to efficient processing of readthrough transcripts from the upstream genes when *trnP* is excised. The *aadA* marker gene probe recognized a 1 kb mRNA in TVV1 through TVV4 lines, as expected (Fig. 1c).

Expression of transgenes in the chloroplasts may interfere with plastid function. Therefore, it was of interest to determine if expression of the *aadA* marker gene adversely affects biomass accumulation. To address this problem, we grew the transplastomic plants for 45 days in a randomized experimental design, then measured plant height and biomass accumulation. A general phenotypic comparison did not reveal any readily identifiable differences between TVV transplastomic lines and wild type. (Fig. 1d). To further discern potential metabolic differences, height and dry weight measurements were taken from pre-bolting mature greenhouse grown plants equally represented and randomly positioned across 8 randomized blocks. Two transplastomic lines, Nt-pMRR15 and Nt-pMRR18, were used as controls and were previously characterized with high level GFP expression of 29.3% and 12.7%, respectively ^26^. A comparison of mean height and dry biomass for each line to wild type shows that only the high levels of recombinant protein expressed in the Nt-pMRR15 plants significantly reduced plant biomass (Fig. 1d).

Inheritance of spectinomycin resistance has been tested in the seed progeny with the unexpected outcome, that expression of spectinomcyin resistance can be most efficiently tested by germinating seed in the absence of sucrose (Supplementary Fig. S1). The *aadA* marker gene has also been excised by introducing the Int site-specific integrase *via* crossing (Supplementary Fig. S2). Introduction of the site-specific recombinase by crossing was less efficient for the removal of the marker gene than direct transformation (Supplementary on line information).

### The expression of Fc fusion proteins in leaves.

**Figure 2.**
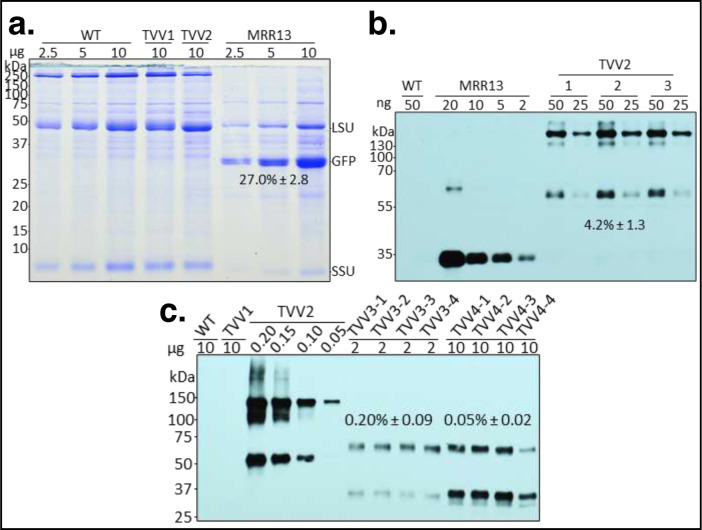
Quantification of GFP and GFP-Fc1 accumulation in transplastomic TVV tobacco leaves. (**a**) Quantification of GFP in the pMRR13 line by 1D-Multilane Densitometry (Alphaimager 2200, ProteinSimple, San Jose, CA, USA). Leaf extracts were separated in SDS-PAGE (12.5%) and stained with Coomassie brilliant blue. (**b**) Quantification of GFP-Fc in the TVV2 line on immunoblots, using GFP Ab and a dilution of pMRR13 extract as reference. GFP-Fc abundance was measured against GFP in the MRR13 reference line (four biological replicates; mean ± 1 SD) with the ImageJ software (version 2.0.0-rc-38/1.50b) (**c**) Quantification of proIGF-I-GGG-Fc1 in the TVV3 line and proIGF-I-2xFurin-Fc1 in TVV3 line on immunoblots, using Fc Ab and a dilution of TVV2 extract as reference. Protein concentrations were calculated with the ImageJ software (version 2.0.0-rc-38/1.50b; four biological replicates; mean ± 1 SD). LSU, rubisco large subunit; SSU, rubisco small subunit.

**Figure 3.**
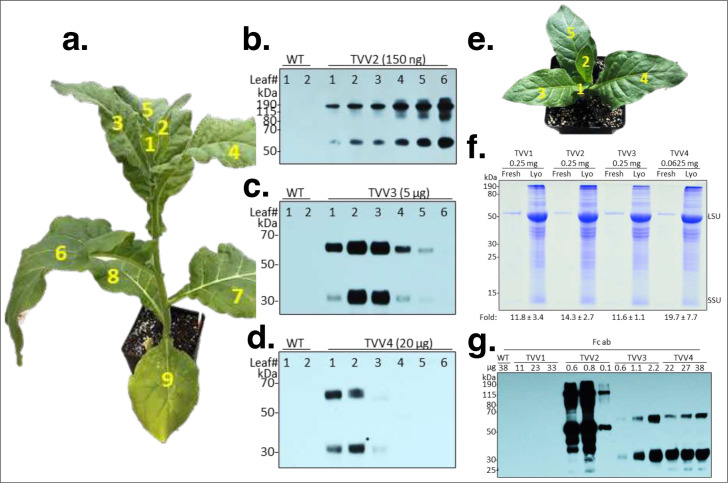
Accumulation of recombinant proteins depends on leaf age and protein stability. (**a**) 40-day old TVV2 transplastomic tobacco plants. Leaves are numbered from top to bottom with the youngest leaf closest to the apical meristem labelled as #1. (**b**) GFP-Fc1 accumulation detected on immunoblots in TVV2 transplastomic leaves. No data are shown for leaves 7, 8 and 9 because the protein extracts of the leaves contained only low protein concentrations. (**c**) proIGF-I-Fc1NC in TVV3 and (**d**) proIGF-I-Fc1C in TVV4 leaves. Protein accumulation was detected on immunoblots, using Fc antibody. (**e**) TVV plant 14 days after cutting back. (**f**) Comparing fresh and lyophilized (Lyo) protein extracts after separation in polyacrylamide gels. Proteins were separated in Bis-Tris MES SDS polyacrylamide gel (12.5%) and stained with Coomassie brilliant blue. Protein content in dried leaf relative to fresh protein extracts was estimated by comparing LSU signal intensity in Coomassie stained gels (three to five replicates; mean ± 1 SD). Fold differences in LSU signal intensity were quantified with ImageJ software (version 2.0.0-rc-38/1.50b). (**g**) Western blot probed with the Fc antibody was used to detect the physical state of lyophilized protein. Western blots of protein extracts from two different lyophilization batches (lyo1, lyo2) are shown.

Accumulation of the GFP-Fc1 protein was assessed in the protein extracts of transplastomic plants (Fig 2a). For consistency, we prepared protein extracts from the second youngest leaf (#2) of the apical meristem (Fig. 3a). The GFP-Fc1 fusion protein was not visible in the Coomassie stained SDS-PAGE gel containing protein extracts of TVV2 plants (Fig. 2a), therefore a source of GFP protein was needed to generate a standard curve for quantification on Western blots. We chose the pMRR13 transplastomic tobacco line known to accumulate high levels of GFP in the leaf total cellular protein (TSP) ^39^. In our hands, the level of GFP in pMRR13 plants was about 27% of TSP (Fig. 2a). Using immunoblots hybridized with the Living Colors primary antibody for the detection of GFP, and MRR13 leaf protein extract as a reference, an average of three biological replicates showed GFP-Fc1 accumulation at 4.24% ± 1.31 of TSP, or 382.47 μg/g fresh leaf material (Fig. 2b). GFP-Fc1 fusion protein displayed two bands, the smaller of which had an electrophoretic mobility consistent with the predicted monomer, and the larger consistent with that of a dimer. Of the two, the dimer was the dominant band in protein samples extracted from young leaves. GFP is monomeric, but Fc forms dimers ^40^, explaining the presence of dimers GFP-Fc1.

Protein extracts of TVV2 plants were the source of reference GFP-Fc protein for quantification proIGF-I-Fc1NC and proIGF-I-Fc1C proteins in TVV3 and TVV4 leaf extracts using the Fc Ab (Fig. 2c). Averages of four biological replicates were calculated for each. Extracts of both types of plants displayed two bands, the smaller of which had an electrophoretic mobility consistent with the predicted protein weight of a monomer (~37 kDa) and the larger consistent with that of a dimer. The leaves accumulated 0.20% ± 0.09 and 0.05% ± 0.02 TSP of proIGF-I-FcNC and proIGF-I-Fc1C, the equivalent of 24.07 μg/g and 5.90 μg/g fresh leaf, respectively (Table 2).

Protein synthesis is active in young, developing leaves and slows down in older leaves. Therefore, unstable proteins are expected to accumulate only in young leaves whereas stable proteins may be present in most leaves. To decide which leaves to harvest for lyophilization we tested protein accumulation in the leaves of plants at the 9-leaf stage before the flower buds appeared (Fig. 3a). TVV2 leaves contained increasing amounts of GFP-Fc1 from the youngest leaf (#1) through the oldest leaf (#6) tested in the fully grown plants (Fig. 3b). In this regards GFP-Fc behaves as FaeG, a highly stable proteinaceous polymer with a capacity to evoke mucosal immune responses ^41^. Recombinant protein proIGF-I-Fc1NC accumulated only in leaves 1–4 in TVV3 plants (Fig. 3c), and proIGF-I-Fc1C in TVV4 plants only in leaves 1 and 2 (Fig. 3d). Therefore, we collected #1 and #2 leaves for lyophilization.

### Lyophilization of bioencapsulated Fc fusion proteins.

Lyophilization of transplastomic leaf tissues protects bioencapsulated recombinant proteins from degradation and enables the storage of desired aliquots or “doses” for murine oral gavage trials ^42,43^. To create a source of rapidly growing young leaves in a short period of time, flowering plants were cut back to the oldest leaf. The plants then were treated with 14:14:14 slow-release fertilizer pellets. Numerous small shoots quickly emerged from lateral buds. After 2 days all shoot buds were removed with the exception of a single one that was allowed to develop to the 5-leaf stage (Fig. 3e). In 14 days the 2^nd^ and 3^rd^ youngest leaves were harvested from 20–25 plants to obtain a total 150 g of fresh leaf material. Freeze-drying 150 g fresh leaf yielded 5 to 9 g dry leaf with an approximate 12% residual water content. Protein-content and quality in fresh and lyophilized leaf material was compared after separating protein extracts in SDS-PAGE followed by Coomassie blue staining. We estimate, based on the signal intensity of the rubisco large subunit, that lyophilization concentrated the protein content on average 15 times (Fig. 3f). Western blots probed with the Fc antibody indicate that the monomeric and dimeric forms of the Fc fusions were preserved during lyophilization and that there was no readily detectable protein degradation during storage (Fig. 3g).

### Bioactivity of Pro-IGF-I-Fc1.

**Figure 4.**
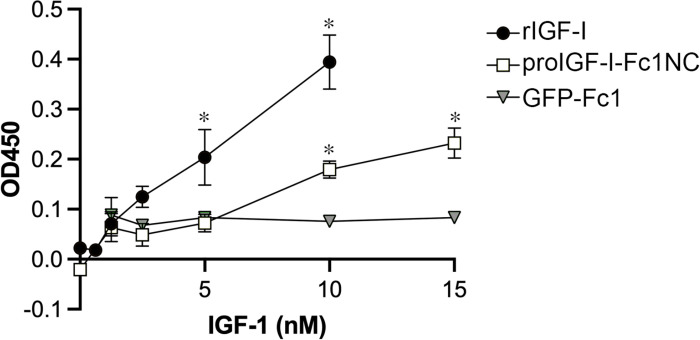
IGF-I receptor activity of proIGF-I-Fc1NC. KIRA assays were performed on protein extracts from plants expressing proIGF-I-Fc1NC or GFP-Fc1 with murine recombinant IGF-I (rIGF-I) serving as a positive control. Data shown is from 1 representative biological replicate, with mean ± SD of technical replicates. GFP-Fc1 is shown as equivalent serial dilutions but contains no IGF-I. *, P<0.05 vs no sample by 1 way ANOVA followed by Dunnett’s multiple comparisons test.

Even though there was insufficient production of either IGF-I sequence to evaluate oral uptake in mice, it enabled evaluation of IGF-I receptor activity in cell-based assays. IGF-IR phosphorylation was significantly increased with exposure to 10nM IGF-I from TVV3 (Fig. 4). Linear regression analysis revealed that equivalent phosphorylation levels were achieved by 12nM TVV3 as 5 nM recombinant IGF-I, and at 10nM for both sources, TVV3 phosphorylation was 45% of recombinant IGF-I. This assay indicated that the IGF-I produced in chloroplasts was remarkably active, even though production was lower than expected.

### Oral uptake and detection in circulation of GFP-Fc in mice.

**Figure 5.**
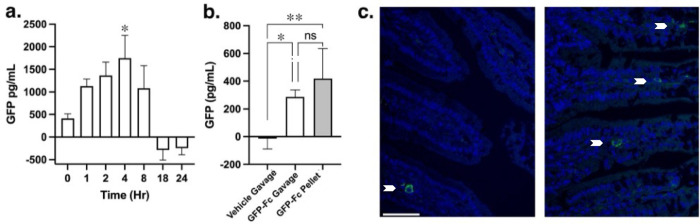
Oral bioavailability of GFP-Fc1 fusion protein expressed in transplastomic tobacco leaves. (**a**) Circulating GFP levels following oral gavage of 20 mg plant material into hFcRn Tg mice (N=5 mice, 3M, 2F). GFP was above background by 1 hr postgavage, peaked at 4 hrs, and returned to baseline by 18 hours. *, P<0.05 vs 0 hr by 1 way ANOVA followed by Dunnett’s multiple comparisons test. (**b**) Circulating GFP 18–20 hours after a 3-day dosing regimen by oral gavage or dough diet. Both methods of GFP-Fc1 delivery were similarly effective in increasing circulating GFP (N=4–6 per condition). *, P<0.05; **, p<0.01 vs vehicle gavage by 1 way ANOVA followed by Tukey’s multiple comparisons test. (**c**) GFP detection in cells within the villi of the mouse jejunum 4 hours after oral gavage delivery of GFP-Fc1. Images are taken from 2 separate mice. Scale bar, 50 μm.

Adult mice expressing the humanized Fc neonatal receptor (hFcRn) provide a relevant model to test oral bioavailability of Fc fusion proteins ^44^. A single dose of GFP-Fc delivered by oral gavage was detectable in circulation as early as 1 hour after delivery, and GFP levels returned to baseline by 18 hours (Fig. 5a). We compared two methods of delivery for a period of 3 days and found that trough levels of GFP in the circulation the following day were similar between oral gavage or dough diet (Fig 5b). Many tissues from the same mice were subjected to GFP ELISA measurements. However, there was no detectable signal found in any of the tissues (data not shown). To determine if oral bioavailability was achieved through transcytosis in the gut, immunostaining for GFP was performed on fixed sections of the jejunum. A subset of cells within the villi were detected with GFP signal surrounding the nucleus (arrow, Fig. 5c), supporting that these cells afforded uptake. Taken together, oral delivery of Fc fusion proteins expressed in the chloroplasts is achievable through gut absorption and paves the way for this strategy to be implemented for therapeutic proteins.

## DISCUSSION

Our goal was high-level expression of the Fc fusion proteins in chloroplasts for testing oral bioavailability. Protein accumulation from transgenes depends on mRNA abundance determined by promoter strength and mRNA stability; the efficiency of translation determined by the 5’-UTR. The plastid rRNA operon PEP promoter (Prrn) is the strongest known promoter in chloroplasts ^45^, which was linked to the maize *psbH* 5’-UTR (LatpH^Zm^) , containing a PPR10 RNA binding protein target site, the most efficiently translated reading frame in chloroplasts ^46,47^. This promoter-leader combination in a dicistronic construct (pAI3) resulted in the accumulation of 23% GFP in potato leaf ^35^. The GFP-Fc protein, expressed from the PrrnLatpH^Zm^ 5’ regulatory region accumulated only at 4.27% of TSP. Protein models of the GFP-Fc1 fusion show low modeling scores at the interconnecting region between the C-terminus of the GFP and the N-terminus of the CH2 domain of Fc ^48^. This region consists of a GGG linker, with the remaining GCKPCICT portion of the Fc hinge, and may represent a region of structural mobility or low structural integrity, both of which may subject the fusion protein to degradation. The non-cleavable proIGF-I-Fc1NC protein (TVV3) and proIGF-I-Fc1 with the cleavable linker (TVV4) accumulated at even lower level, 0.2% and 0.05% TSP. Reduced proIGF-I-Fc1 in TVV4 transgenic lines can be attributed to faster protein degradation due to differences of amino acid sequences in the linker region.

After conversion of 4.27 % TSP to μg/g fresh weight, protein accumulation of GFP-Fc1 measured approximately 0.3825 μg/mg of fresh weight and, after lyophilization, concentrated to 5.45 μg/mg of dry weight. These results are comparable to those obtained in previous reports where accumulation of CTB-GFP, PTD-GFP and DCpep-GFP in lyophilized transplastomic tobacco tissues, measured against a standard curve of purified GFP, was 5.6 μg/mg, 24.1 μg/mg and 2.16 μg/mg of dry weight, respectively ^49^. The use of a purified, commercially available GFP as reference protein was also our first choice for quantification of the GFP-Fc1 fusion, however this method inflated accumulation measurements almost 9-fold. Heterologous protein expression at these levels should produce a heavy band in the Coomassie gels and a metabolic burden on the transplastomic plants, as our previous experience shows ^26^ but neither of which occurred here. We found greater accuracy using the previously characterized MRR13 transplastomic tobacco line as a reference for measuring GFP-Fc1 accumulation.

Protein accumulation of proIGF-I-Fc1 in TVV3 and TVV4 was 24.07 μg/g and 5.90 μg/g of fresh weight, respectively. Lyophilization concentrated the protein to 279.9 μg/g of dry weight in TVV3 and 116.0 μg/g of dry weight in TVV4. These results are comparable to those obtained in previous reports where accumulation of CTB-ProIGF-I and PTD-ProIGF-I in lyophilized transplastomic lettuce tissues was 370 μg/g and 270 μg/g of dry weight, respectively ^10^. The difference in protein accumulation in the fresh tissues of TVV3 and TVV4 lines is likely due to differences in protein stability due to structural variations in the linker region, as discussed above.

We report here new TVV vectors with significant improvement over our currently available chloroplast expression vectors. (i) The spectinomycin resistance marker *aadA* is low impact, because it does not reduce the biomass of transplastomic plants (Fig 1d). We previously reported that the expression of spectinomycin resistance (*aadA*) marker gene in line Nt-pMRR15 significantly reduces biomass accumulation ^26^. We also reported that expression of *aadA* in a PrrnLatpB promoter / T*his*/*thr* terminator cassette reduced biomass by about 10%, and that excision of marker gene restored wild-type biomass levels ^50^. (ii) Readthrough transcription can generate multiple RNA species from the transgenes producing complex patterns on RNA gel blots, see for example ^51^. Inclusion of a tRNA between the marker gene and the gene-of-interest facilitates processing of readthrough transcripts. Indeed, RNA gel blots of plants obtained with the TVV vectors reveal complete processing of mRNA transcripts, simplifying quantitative evaluation of mRNA accumulation (Fig. 1c). (iii) Inclusion of PhiC31 recombinase target sites enable convenient post-transformation removal of the marker genes, as documented here. Transformation of the nuclear genome of T0 transplastomic plants typically results in excision of the marker gene in 100% of plastid genomes by the time a shoot regenerates and DNA gel blot analyses can be performed on the leaves. That is why we were surprised to find partial excision in each of the F1 progeny of TVV plants obtained by pollination with three independent nuclear transgenic lines carrying the Int gene ^50^. Partial excision of the marker gene in the F1 generation is probably due to inactivity of the Agrobacterium 2’ promoter driving Int expression in the female gametocyte, a problem that can be avoided by using female gametocyte-specific promoters.

Administering biopharmaceuticals by injection has disadvantages, including the need for refrigerated storage and transportation, and the requirement for skilled personnel to administer the medication. Oral delivery of these drugs could eliminate the need for refrigerated storage and transportation and would revolutionize healthcare. A major challenge associated with oral delivery is degradation of biopharmaceuticals by acids and proteases that can be avoided by bioencapsulating proteins the within plant cells in chloroplasts. In the gut, commensal microbes break down the cell wall and release the protein drug into the intestinal lumen from where it should get into the immune or circulatory system. Translocating the released protein requires fusing it with a transmucosal carrier protein.

In the early experiments on plant-made pharmaceuticals, no fusion protein was available for testing. Testing of the non-toxic Tet-C fragment of tetanus toxin was accomplished by mixing tobacco leaf extracts with a small amount of cholera toxin (CT) and applied to the nasal surfaces of mice. CT acted as an adjuvant, and immunization with the leaf extract provided protective immunity to the treated mice ^25^.

In the meantime, a number of mucosal carrier proteins have been tested as carriers of plant-made pharmaceuticals. Cholera toxin B (CTB) is the most used fusion partner in chloroplasts ^9^. CTB binds GM1 receptors on cell membranes and facilitates delivery of fusion proteins through endocytosis. Protein delivery was first shown using a CTB-GFP fusion protein. In addition to CTB, fusion of the GFP C-terminus with the protein transduction domain (PTD), a small cationic human peptide that can penetrate cell membranes without specific receptors and a dendritic cell peptide (DCpep), were shown to deliver GFP orally ^10,49^.

We report here the GFP-Fc fusion protein for oral delivery. Examples of Fc-fusion proteins made in plants have been published, including human prostatic acid phosphatase ^52^, cocaine hydrolase-Fc ^53^, osteopontin-Fc fusion protein ^54^ and a plant-expressed Fc-fusion protein tetravalent dengue vaccine ^55^. These proteins have been expressed from nuclear genes and targeted for secretion via the ER, where Fc is glycosylated. One consideration in using Fc-fusion proteins is the role of glycosylation in modulating Fc receptor binding and specificity. The Fc Asn 297 is glycosylated but is within the core of the Fc dimer. Non-glycosylated Fc remains stable and folds properly, but the dimer formed by Fc domains lacking Asn 297 glycosylation has a tighter conformation, and this can reduce FcRn binding by ~10 fold ^56^. Plant and metazoan glycosylation differ ^57^, leading to possibly diminished FcRn binding and absorption. Glycosylation is absent in chloroplasts. We expect that, when high level chloroplast expression is obtained, high level expression compensates for reduced stability caused by the lack of glycosylation. This is particularly relevant in case of proIGF-Fc, because proIGF is more active when not glycosylated. The answers to these open questions will be delivered by future experimentation.

## MATERIALS AND METHODS

### Plant material and growth conditions.

Experiments were carried out using *Nicotiana tabacum* L. cv. Petit Havana plants. The tobacco seed was a gift of Canada Department of Agriculture, Ottawa Research Station to the Institute of Genetics, Hungarian Academy of Sciences, Budapest. PM received the seed from the institutional stock in 1970 and maintains it since. Seed is also available from Lehle Seed, Round Rock, TX 78681, United States. Seed is available for free and without any restriction upon request to PI, after paying for postage.

Seedlings were germinated under aseptic conditions from surface-sterilized seeds under 16h illumination at 28°C using cool-white, fluorescent tubes (2,000 lx, CXL F025/741), followed by an 8h dark period at 21°C. Seeds germinated under greenhouse conditions were illuminated for 16 hrs.

### Research with tobacco plants.

Experimental research with tobacco plants (*Nicotiana tabacum*.cv Petit Havana) was carried out in compliance with the relevant institutional and national guidelines and regulations, according to Biosafety Protocol entitled “Expression of Foreign Genes in Plants” (IBC Registration #: 12–089), approved by the Rutgers University Institutional Biosafety Committee.

### Plastid transformation vector construction.

The vector backbone in which all subsequent vectors were created is pPRV1-II ^58^, a PUC119/pZS192 derivative ^59^ containing *N. tabacum* plastid sequences to facilitate homologous recombination between the plastid *trnV* gene and the *3’rps12/7* promoter. TVV1 carries the spectinomycin resistance (*aadA*) ^37^ selection marker with a C-terminal *c-Myc* tag facilitating detection of AAD on protein gel blots. The selectable marker is expressed in a *rbcL* promotor ^60^ and *psbA* terminator ^61^ cassette in which the *trnP* from the *Medicago trunculata* plastid genome (AC093544 ) is incorporated between the multiple cloning site and the *aadA-cMyc* gene to facilitate efficient cleavage of potential readthrough transcripts ^32,33^. The resistance marker is also flanked by minimal *attB* (34 bp) /*attP* (39 bp) sites ^31^ for post-transformation excision by the PhiC31 phage-site-specific integrase ^62^. Vector TVV2 contains the *eGFP-Fc1* fusion gene with a non-cleavable 3x glycine linker; TVV3 carries a *proIGF-I-Fc1* fusion with a non-cleavable 3x glycine linker; TVV4 carries a *proIGF-I-Fc1* fusion with a cleavable 2x furin linker; TVV5 carries the *myostatin propeptide-Fc1* fusion. The genes of interest in TVV2, TVV3, TVV4, and TVV5 are expressed from the tobacco plastid ribosomal RNA PEP promoter (*Prrn*) ^63^ fused with the *Zea maize atpH* 5’-UTRs, referred to as leader sequences (L)^46^. Termination is controlled by a histidine-threonine attenuator. TVV1 was generated through a single insertion into the *Sca*I site of pPRV1-II and the TVV2, TVV3, TVV4, and TVV5 were cloned as a *Sac*I*-Hind*III fragment into the multiple cloning site of TVV1. The schematic map of the vectors is shown in Fig. 1a. DNA sequences are listed in Supplemental Table S1.

### Transformation of the tobacco plastid genome.

Tobacco plastid transformation was carried out as described ^58,64^. Briefly, a Bio-Rad PDS-1000 biolistic gun and Hepta Adaptor was used to bombarded sterile tobacco leaves with 0.6 μm gold particles coated in vector DNA. Leaves transferred to selective RMOP shoot regeneration medium containing 500 mg/L spectinomycin form pigment deficient callus. Transplastomic events express the selective marker gene and emerge as green shoots from calli. Primary events were subjected to one or two additional rounds of regeneration on spectinomycin medium to dilute out residual wild-type copies of the plastid genome Transplastomic events are distinguished from spontaneous spectinomycin resistant mutants by streptomycin-spectinomycin resistance while spontaneous spectinomycin resistant mutants are sensitive to streptomycin. Transgene integration into the plastid genome is confirmed by Southern blot analyses. In genetically stable homoplastomic plants all wild type chloroplast genome copies are absent. The shoots were rooted on hormone-free medium and transferred to the greenhouse.

### PCR analysis.

PCR was used for the initial identification of transplastomic events (T_0_). The forward primer, LTRF4 5’-CACCACGTCAAGGTGACACT-3,’ is in the *trnV* gene of the left targeting region of the chloroplast genome target site. The reverse primer, aadAR2 5’-GTTGAGTCGATACTTCGGCG-3,’ is in the *aadA* coding region. Expected band sizes for transplastomic events are as follows: 0.5 kb for TVV1, 2.3 kb for TVV2, 1.9 for TVV3, 2.0 kb for TVV4, and 2.3 kb for TVV5. A single *Ear*I restriction digest site within the TVV4 amplicon allows for further discernment from TVV3. Table 3 contains primers used to verify transplastomic events, generate probes, and analyze marker excision.

### Compliance with the digital image and integrity policies.

Where gels/blots are used in figures, we have checked their compliance with the digital image and integrity policies of the journal (https://www.nature.com/srep/journal-policies/editorial-policies#digital-image).

### Southern blot analysis.

DNA gel blot analysis was carried out as described^37^. Briefly, the cetyltrimethylammonium bromide (CTAB) protocol was used to extract total cellular DNA from leaf tissue. Two micrograms of total leaf DNA was digested with the *Eco*RI restriction endonuclease followed by separation on 1% agarose gels. The DNA was then transferred to Hybond-N membranes (GE Healtcare RPNBL/02/10) by capillary blotting. Two probes, the plastid *rrn16S* and *aadA,* were prepared by random-primed ^32^P labeling using the Takara Bio. Inc., kit (Cat. No. 6045). The *rrn16S* probe was the 1.9 kb *Apa*I/*Bam*HI ptDNA fragment encoding part of the 16S rRNA gene, while the aadA probe was a 0.7-kb fragment amplified from the *aadA* coding region using primers *aada* Fw 5’-GTTGCTGGCCGTACATTTG-3’ and *aadA* Rv 5’-TCGCCTTTCACGTAGTGGAC-3’.

### Northern blot analysis.

Leaves were frozen in liquid nitrogen and total cellular RNA was isolated using the Qiagen RNeasy Plant Mini Kit (Cat. No. 74904). The RNA was separated in a 1.5% formaldehyde gel and transferred to Hybond-N membranes by capillary blotting (GE Healthcare RPNBL/02/10). The probes were: *aadA*, the same which was used for Southern blot analysis, and *Fc1,* a 0.6-kb fragment amplified from the *Fc1* coding region using primers *Fc1-*F1 5’-ATGCAAACCCTCGTGCAGTA-3’ and Fc1-R1 5’- GCAAGCCCTGTATTTGTACGG-3’. The probes were prepared by random-primed ^32^P-labeling using the Takara Bio. Inc., kit (Cat. No. 6045).

### SDS-PAGE and immunoblot analysis.

Leaves were harvested from plants grown under greenhouse conditions and frozen in liquid nitrogen. To obtain total soluble protein (TSP), about 100 mg of leaf material was pulverization with stainless steel grinding balls (SPEX Sample Prep, Metuchen, NJ) and suspended in 100 μl of extraction buffer containing 50 mM HEPES-KOH (pH 7.5), 10 mM potassium acetate, 5 mM magnesium acetate, 1 mM EDTA, 1 mM dithiothreitol, 2 mM phenylmethylsulfonyl fluoride, and protease inhibitor cocktail (30 μl/mL) (Sigma-Aldrich, Cat. No. P9599). The suspension was centrifuged at 14,000g for 5 min at 4°C and the supernatant was transferred to a new Eppendorf tube. This step was repeated once more. To analyze lyophilized material, 10 mg of lyophilized leaf power was rehydrated in 100 μL of extraction buffer. Protein concentration was determined with the Bio-Rad Bradford Protein Assay (Cat. No. 5000002). The proteins were separated in urea PAGE or Bis-Tris PAGE before transfer to an Immun-Blot PVDF membrane (Bio-Rad, Cat. No. 1620177). Detection of EGFP required the Living Colors^®^ A.v. Monoclonal Antibody (JL-8) (Takara, 632381) as the primary antibody and the AntiMouse IgG Peroxidase antibody (Sigma-Aldrich, A4416-1) as the secondary antibody. For the detection of Fc1, the Anti-Mouse IgG1 Rabbit Monoclonal Antibody (RevMAb Biosciences, 31-1002-00) was used as the primary antibody and the Goat Anti-Rabbit IgG Peroxidase antibody (Sigma-Aldrich, A0545-1) as the secondary antibody. Detection of the AAD-cMyc tag required the c-Myc Antibody (9E10) (Santa Cruz Biotechnology, sc-40) as the primary antibody and the Anti-Mouse IgG Peroxidase antibody (Sigma-Aldrich, A4416) as the secondary antibody.

### Lyophilization.

Plants grown to the 9-leaf stage were cut down to the basal node and regenerated to the 5-leaf stage. The second and third leaves from the top were harvested from the regenerated plants, washed in soapy water, and rinsed 5 times before removal of the central vein and subsequent slicing into 1–2 cm^2^ pieces. 150 g of leaf material was placed in a tray and frozen at −80°C before being lyophilized in the VirTis Genesis 25EL Pilot Lyophilizer vacuum (10 mTorr) at −50°C for 48 hours, −30°C for 48 hours, −20°C for 48 hours, −15°C for 48 hours, −10°C for 48 hours, and 15°C for 24 hours. Moisture content was checked with the Mettler Toledo infrared moisture analyzer and the final moisture content, representing water molecules tightly bound to cellular compounds, was approximately 12%. Lyophilized material was stored in 8 Oz. Glass Mason Jars (Ball, Quilted Crystal Regular Mouth Half-Pint) with a 5 g silica gel desiccant packet (LotFancy, 12M-2586-L) and sealed with a vacuum sealer (FoodSaver^®^ FM3600 series and Regular Jar Sealing Accessory).

### Biomass assay.

T_1_ seed was germinated under greenhouse conditions and transferred two times: first to 1”×2” cells and then to 4”×4” pots. The larger pots were situated in a randomized block design of 8 blocks where a table represented a block containing 14 randomized plants: two individuals from each of the 4 successful vector lines, two wild type plants, and two plants from 2 control lines previously characterized ^65^: pMRR15, and pMRR18. Height and biomass data was collected when the flower buds appeared. Height was measured from the base of the soil line to the base of leaf no. 1 (Fig. 3a, e). Then the plants were cut down at the soil line and the entire shoot was placed in a paper bag and dried in an oven at 80°C for 5 days. Fresh weight and dry weight measurements were taken before and after drying.

### Mouse studies.

Animal studies were approved by the University of Florida Institutional Animal Care and Use Committee (IACUC), and the experiments were performed in accordance to relevant guidelines and regulations. In addition, animal studies were reported in accordance with ARRIVE guidelines. Male and female mice with ablation of murine FcRn and transgenic expression of the human FcRn regulated by the native human promoter (FcRn^−/−^hFcRn (32)Tg, Jax #014565) ^44^ were utilized. Oral bioavailability of GFP-Fc1 fusion proteins was evaluated in male and female hFcRn Tg mice (all age 10 –14 weeks). Initial testing used 20mg lyophilized plant TVV2 expressing GFP-Fc1 suspended in 200uL PBS as a single bolus delivered by oral gavage after a 4 hour fast (N=5). Blood was collected from the facial vein prior to gavage, and at 1, 2, 4, 8, 18, and 24 hours after oral gavage. Blood was centrifuged to collect serum for GFP quantification and stored at −80C until analysis.

Subsequent testing compared oral gavage to ingesting lyophilized plant mixed into a dough diet (Bioserv Transgenic Dough Diet (S3472)). The oral gavage procedure was performed as described above for 3 days using TVV1 (vehicle) (N=4) or TVV2 (GFP-Fc1) (N=8). For the dough diet, mice were familiarized to the diet provided in 300 mg pellets including 20mg TVV1 plant material for 5 days. The following week, mice were given TVV2 (15 mg TVV2/300 mg dough, 1 pellet/day) for 3 days (N=6), with N=2 mice receiving no treatment. For all groups, mice were euthanized 18–20 hours after the final dose using exposure to carbon dioxide followed by cervical dislocation in accordance with American Veterinary Medical Association’s (AVMA) Guidelines for the Euthanasia of Animals. Blood, brain, heart, liver, kidney, small intestine, and skeletal muscles (diaphragm, soleus, tibialis anterior, quadriceps and extensor digitorum longus) were collected, rapidly frozen in liquid nitrogen, and stored in −80C for GFP quantification.

### GFP Detection in small intestine.

hFcRn Tg mice were subjected to a single dose of TVV2 by oral gavage as described above and were euthanized 4 hours after TVV2 delivery. Following euthanasia, the jejunum of the small intestine was removed, flushed with PBS, and then fixed overnight in 4% paraformaldehyde at 4C. Tissues were incubated serially in 10% and 20% sucrose, surrounded in optimal cutting temperature compound (Sakura, Torrance, CA), and frozen in liquid nitrogen-cooled isopentane for histological analysis. 10 μm cryosections were subjected to immunostaining for GFP. Sections were washed in PBS 3 times, with 10 minutes each wash, followed by permeabilization in 0.5% Triton-X in PBS. Following blocking in 5% bovine serum albumin (BSA) in PBS for 1 hour at room temperature, sections were incubated overnight at 4°C with primary antibodies diluted in 5% BSA for GFP (1:3000 rabbit pAb anti-GFP, Cat#ab6556, Abcam). After washing slides in PBS 3 times, with 10 minutes each wash, tissue autofluorescence was quenched with 0.1% Sudan Black incubation with subsequent washes. Next, sections were incubated for 1 hour at room temperature in the dark in secondary antibodies diluted in 5% BSA (1:1000 Alexafluor 568 IgG anti-rabbit (#A11036) (Invitrogen)). Sections were washed again in the dark (PBS 3 times, with 10 minutes each wash.), then airdried, and covered with mountant (ProLongTM Diamond Antifade with Dapi, Cat#P36962, ThermoFisher) and cover slip. Samples were visualized with a Leica STELLARIS confocal microscope (Leica Microsystems, Buffalo Grove, IL, USA). Images were acquired and processed with the Leica Application Suite and Microscope Imaging software (Leica Microsystems).

### IGF-IR activation Assay.

To evaluate the potency of IGF-IR activation by IGF-I produced by TVV3 plants, a kinase receptor assay (KIRA) was performed as previously described . Briefly, 2.5 × 10^4^ P6 cells, which overexpress IGF-IR (kind gift from Dr Renato Baserga, Thomas Jefferson University, Philadelphia, Pennsylvania) were seeded into 96-well plates. They were maintained in growth media supplemented with 200 μg/mL G418. The cells were serum starved for 6 hours, and then treated for 15 min with protein extracts obtained from lyophylized plants. Controls included P6 cells treated with protein extracts from GFP-Fc1/TVV2, or with recombinant IGF-I (0.5–10 nM). The P6 cells were lysed and IGF-IR was captured onto an ELISA plate coated with an antibody to IGF-IR (MAB1120, Millipore Corp.). A horseradish peroxidase-conjugated antibody to phosphorylated tyrosines (16–454, Millipore Corp.) and TMB substrate (N301, Thermo Scientific, Rockford, Illinois) were used for colorimetric quantification. Absorbance was read at 450 nm via the SpectraMax M5 plate reader (Molecular Devices, Sunnyvale, California). Measurements were done in technical triplicates, and performed in entirety twice.

## Figures and Tables

**Table 1.** Frequency of streptomycin resistant / spectinomycin resistant transplastomic lines among spectinomycin resistant clones

Vector	No. plates	No. Spec^R^	Tested	Strep^R^/Spec^R^	% Double Resistant

pTVV1	3	93	14	11	79%
pTVV2	3	26	12	4	33%
pTVV3	3	33	19	14	74%
pTVV4	3	46	15	11	73%
pTVV5	6	19	19	3	16%

**Table 2.** Accumulation of recombinant proteins (% TSP) in TVV leaves. MRR13 is the source of reference GFP protein. N = number of leaf samples. F.w. = fresh weight

Plant line	Protein	N	%TSP	SD	%TSP + SD	μ/g F.w. ± SD

MRR13–1	GFP	6	27.12	1.70	26.98 ± 2.78	
MRR13–2	GFP	6	26.34	3.30		
MRR13–3	GFP	6	27.47	3.32		

TVV2–1	GFP-Fc	14	3.36	1.23	4.24 ± 1.40	382.47 ± 68.49
TVV2–2	GFP-Fc	14	5.04	1.54		
TVV2–3	GFP-Fc	14	4.31	1.41		

TVV3–1	proIGF-I-Fc1NC	8	0.17	0.07	0.20 ± 0.10	24.07 ± 0.99
TVV3–2	proIGF-I-Fc1NC	8	0.20	0.09		
TVV3–3	proIGF-I-Fc1NC	8	0.17	0.09		
TVV3–4	proIGF-I-Fc1C	8	0.21	0.12		

TVV4–1	proIGF-I-Fc1C	8	0.04	0.02	0.05 ± 0.02	5.9 ± 0.31
TVV4–2	proIGF-I-Fc1C	8	0.04	0.01		
TVV4–3	proIGF-I-Fc1C	8	0.05	0.02		

**Table 3.** Primers used to verify transplastomic events, generate probes, and analyze marker excision.

Primer	Sequence (5’ to 3’)	
eGFP F	TTGATGGGGATGTCAACGGG	651 bp *eGFP* amplicon.
eGFP R	CGTCCATCCCAAGCGTGATA	
aadA Fw	CGTTGCTGGCCGTACATTTG	688 bp *aadA* amplicon to be used as a probe.
aadA Rv	TCGCCTTTCACGTAGTGGAC	
proIGF F	TGCTGAACTGGTTGACGCTT	a 234 bp proIGF1 amplicon.
proIGF R	TGCGTTTTAGGCATGTCTGTG	
TrnP Fw2	CCTGTCATCCCTATCCCTAACTTG	Marker excision is 344 bp. No marker excision is 1,494 bp.
rps12/7R	CCTGCTAGGCAAGAGGATAGC	
LTRF4	CACCACGTCAAGGTGACACT	TVV1–547 bp, TVV2– 2,331 bp, TVV3– 1,923 bp, TVV4– 2,953 bp, TVV5– 2,337 bp.
aadAR2	GTTGAGTCGATACTTCGGCG	
Fc1-F1	ATGCAAACCCTCGTGCAGTA	620 bp *Fc1* amplicon to be used as a probe.
Fc2-R1	GCAAGCCCTGTATTTGTACGG	
AM63	TCTTCCGTGCCGTCCTG	~800 bp *integrase* amplicon.
AM65	CGAGCCCGCTGAGTGG	

## Data Availability

The datasets used and/or analyzed during the current study available from the corresponding author on reasonable request

## References

[1] Mak R. H. & Rotwein P. Myostatin and insulin-like growth factors in uremic sarcopenia: the yin and yang in muscle mass regulation. Kidney Int 70, 410–412 (2006). 10.1038/sj.ki.5001622 16871252

[2] Jones J. I. & Clemmons D. R. Insulin-like growth factors and their binding proteins: biological actions. Endocr Rev 16, 3–34 (1995). 10.1210/edrv-16-1-3 7758431

[3] Lee S. J. Regulation of muscle mass by myostatin. Annu Rev Cell Dev Biol 20, 61–86 (2004). 10.1146/annurev.cellbio.20.012103.135836 15473835

[4] McPherron A. C., Lawler A. M. & Lee S. J. Regulation of skeletal muscle mass in mice by a new TGF-beta superfamily member. Nature 387, 83–90. (1997). 9139826 10.1038/387083a0

[5] Friedlander A. L. One year of insulin-like growth factor I treatment does not affect bone density, body composition, or psychological measures in postmenopausal women. J Clin Endocrinol Metab 86, 1496–1503 (2001). 10.1210/jcem.86.4.7377 11297574

[6] Heatwole C. R. Open-label trial of recombinant human insulin-like growth factor 1/recombinant human insulin-like growth factor binding protein 3 in myotonic dystrophy type 1. Arch Neurol 68, 37–44 (2011). 10.1001/archneurol.2010.227 20837825 PMC3374954

[7] Lai E. C. Effect of recombinant human insulin-like growth factor-I on progression of ALS. A placebo-controlled study. The North America ALS/IGF-I Study Group. Neurology 49, 1621–1630 (1997). 10.1212/wnl.49.6.1621 9409357

[8] Philippou A. & Barton E. R. Optimizing IGF-I for skeletal muscle therapeutics. Growth Horm IGF Res 24, 157–163 (2014). 10.1016/j.ghir.2014.06.003 25002025 PMC4665094

[9] Daniell H., Chan H. T. & Pasoreck E. K. Vaccination via Chloroplast Genetics: Affordable Protein Drugs for the Prevention and Treatment of Inherited or Infectious Human Diseases. Annu Rev Genet 50, 595–618 (2016). 10.1146/annurev-genet-120215-035349 27893966 PMC5496655

[10] Park J. Oral delivery of novel human IGF-1 bioencapsulated in lettuce cells promotes musculoskeletal cell proliferation, differentiation and diabetic fracture healing. Biomaterials 233, 119591 (2020). 10.1016/j.biomaterials.2019.119591 31870566 PMC6990632

[11] Durzynska J. & Barton E. IGF expression in HPV-related and HPV-unrelated human cancer cells. Oncol Rep 32, 893–900 (2014). 10.3892/or.2014.3329 25018100 PMC4121407

[12] Barton E. R. The ABCs of IGF-I isoforms: impact on muscle hypertrophy and implications for repair. Appl Physiol Nutr Metab 31, 791–797 (2006). 10.1139/h06-054 17213901

[13] Brisson B. K. & Barton E. R. Insulin-like growth factor-I E-peptide activity is dependent on the IGF-I receptor. PLoS One 7, e45588 (2012). 10.1371/journal.pone.0045588 23029120 PMC3448668

[14] Wallis M. New insulin-like growth factor (IGF)-precursor sequences from mammalian genomes: the molecular evolution of IGFs and associated peptides in primates. Growth Horm IGF Res 19, 12–23 (2009). 10.1016/j.ghir.2008.05.001 18571449

[15] Durzynska J., Philippou A., Brisson B. K., Nguyen-McCarty M. & Barton E. R. The pro-forms of insulin-like growth factor I (IGF-I) are predominant in skeletal muscle and alter IGF-I receptor activation. Endocrinology 154, 1215–1224 (2013). 10.1210/en.2012-1992 23407451 PMC3578996

[16] Carlsson-Skwirut C., Lake M., Hartmanis M., Hall K. & Sara V. R. A comparison of the biological activity of the recombinant intact and truncated insulin-like growth factor 1 (IGF-1). Biochim Biophys Acta 1011, 192–197 (1989). 10.1016/0167-4889(89)90209-7 2469478

[17] Gray A. M. & Mason A. J. Requirement for activin A and transforming growth factor--beta 1 pro-regions in homodimer assembly. Science 247, 1328–1330 (1990). 10.1126/science.2315700 2315700

[18] Harrison C. A., Al-Musawi S. L. & Walton K. L. Prodomains regulate the synthesis, extracellular localisation and activity of TGF-beta superfamily ligands. Growth Factors 29, 174–186 (2011). 10.3109/08977194.2011.608666 21864080

[19] Walker R. G. Structural basis for potency differences between GDF8 and GDF11. BMC Biol 15, 19 (2017). 10.1186/s12915-017-0350-1 28257634 PMC5336696

[20] Hopkins D. R., Keles S. & Greenspan D. S. The bone morphogenetic protein 1/Tolloid-like metalloproteinases. Matrix Biol 26, 508–523 (2007). 10.1016/j.matbio.2007.05.004 17560775 PMC2722432

[21] Wolfman N. M. Activation of latent myostatin by the BMP-1/tolloid family of metalloproteinases. Proc Natl Acad Sci U S A 100, 15842–15846 (2003). 10.1073/pnas.2534946100 14671324 PMC307655

[22] Daniell H., Ruiz G., Denes B., Sandberg L. & Langridge W. Optimization of codon composition and regulatory elements for expression of human insulin like growth factor-1 in transgenic chloroplasts and evaluation of structural identity and function. BMC Biotechnol 9, 33 (2009). 10.1186/1472-6750-9-33 19344517 PMC2678116

[23] Kavran J. M. How IGF-1 activates its receptor. Elife 3 (2014). 10.7554/eLife.03772 PMC4381924 25255214

[24] McBride K. E. Amplification of a chimeric *Bacillus* gene in chloroplasts leads to an extraordinary level of an insecticidal protein in tobacco. Biotechnology 13, 362–365 (1995). 9634777 10.1038/nbt0495-362

[25] Tregoning J. Expression of tetanus toxin fragment C in tobacco chloroplasts. Nucleic Acids Research 31, 1174–1179 (2003). 12582236 10.1093/nar/gkg221 PMC150239

[26] Yu Q., Tungsuchat-Huang T., Verma K., Radler M. R. & Maliga P. Independent translation of ORFs in dicistronic operons, synthetic building blocks for polycistronic chloroplast gene expression. Plant J 103, 2318–2329 (2020). 10.1111/tpj.14864 32497322

[27] Oey M., Lohse M., Kreikemeyer B. & Bock R. Exhaustion of the chloroplast protein synthesis capacity by massive expression of a highly stable protein antibiotic. Plant J 57, 436–445 (2009). https://doi.org:TPJ3702 [pii] 10.1111/j.1365-313X.2008.03702.x 18939966

[28] Dumont J. A. Prolonged activity of a recombinant factor VIII-Fc fusion protein in hemophilia A mice and dogs. Blood 119, 3024–3030 (2012). 10.1182/blood-2011-08-367813 22246033 PMC3953019

[29] Gan Y. GLP-1-Exendin-4/IgG4 (Fc) fusion protein as a novel drug for diabetes treatment. Exp Clin Endocrinol Diabetes 123, 371–375 (2015). 10.1055/s-0035-1548797 25962403

[30] Rattanapisit K., Srifa S., Kaewpungsup P., Pavasant P. & Phoolcharoen W. Plant-produced recombinant Osteopontin-Fc fusion protein enhanced osteogenesis. Biotechnol Rep (Amst) 21, e00312 (2019). 10.1016/j.btre.2019.e00312 30847284 PMC6389792

[31] Groth A. C., Olivares E. C., Thyagarajan B. & Calos M. P. A phage integrase directs efficient site-specific integration in human cells. Proc Natl Acad Sci U S A 97, 5995–6000 (2000). 10.1073/pnas.090527097 10801973 PMC18547

[32] Canino G. Arabidopsis encodes four tRNase Z enzymes. Plant Physiol 150, 1494–1502 (2009). 10.1104/pp.109.137950 19411372 PMC2705019

[33] Kruszka K. Plant dicistronic tRNA-snoRNA genes: a new mode of expression of the small nucleolar RNAs processed by RNase Z. EMBO J 22, 621–632 (2003). 10.1093/emboj/cdg040 12554662 PMC140725

[34] Puigbo P., Guzman E., Romeu A. & Garcia-Vallve S. OPTIMIZER: a web server for optimizing the codon usage of DNA sequences. Nucleic Acids Res 35, W126–131 (2007). 10.1093/nar/gkm219 17439967 PMC1933141

[35] Yu Q., Barkan A. & Maliga P. Engineered RNA-binding protein for transgene activation in non-green plastids. Nat Plants 5, 486–490 (2019). 10.1038/s41477-019-0413-0 31036913

[36] Liere K. & Maliga P. *In vitro* characterization of the tobacco *rpoB* promoter reveals a core sequence motif conserved between phage-type plastid and plant mitochondrial promoters. Embo Journal 18, 249–257 (1999). 9878067 10.1093/emboj/18.1.249 PMC1171119

[37] Svab Z. & Maliga P. High-frequency plastid transformation in tobacco by selection for a chimeric aadA gene. Proc Natl Acad Sci U S A 90, 913–917 (1993). 10.1073/pnas.90.3.913 8381537 PMC45780

[38] Cotton T. R. Structure of the human myostatin precursor and determinants of growth factor latency. EMBO J 37, 367–383 (2018). 10.15252/embj.201797883 29330193 PMC5793801

[39] Yu Q. G., Tungsuchat-Huang T., Verma K., Radler M. R. & Maliga P. Independent translation of ORFs in dicistronic operons, synthetic building blocks for polycistronic chloroplast gene expression. Plant Journal 103, 2318–2329 (2020). 10.1111/tpj.14864 32497322

[40] Liu L. M. Pharmacokinetics of monoclonal antibodies and Fc-fusion proteins. Protein & Cell 9, 15–32 (2018). 10.1007/s13238-017-0408-4 28421387 PMC5777971

[41] Kolotilin I. Production of a Subunit Vaccine Candidate against Porcine Post-Weaning Diarrhea in High-Biomass Transplastomic Tobacco. Plos One 7 (2012). https://doi.org:ARTN e42405 10.1371/journal.pone.0042405 PMC3411772 22879967

[42] Kwon K. C. & Daniell H. Low-cost oral delivery of protein drugs bioencapsulated in plant cells. Plant Biotechnol J 13, 1017–1022 (2015). 10.1111/pbi.12462 26333301 PMC4769795

[43] Hoelscher M. High-level expression of the HIV entry inhibitor griffithsin from the plastid genome and retention of biological activity in dried tobacco leaves. Plant Mol Biol 97, 357–370 (2018). 10.1007/s11103-018-0744-7 29948657 PMC6061503

[44] Petkova S. B. Enhanced half-life of genetically engineered human IgG1 antibodies in a humanized FcRn mouse model: potential application in humorally mediated autoimmune disease. Int Immunol 18, 1759–1769 (2006). 10.1093/intimm/dxl110 17077181

[45] Shiina T., Allison L. & Maliga P. *rbcL* transcript levels in tobacco plastids are independent of light: reduced dark transcription rate is compensated by increased mRNA stability. Plant Cell 10, 1713–1722 (1998). 9761797 10.1105/tpc.10.10.1713 PMC143938

[46] Pfalz J., Bayraktar O. A., Prikryl J. & Barkan A. Site-specific binding of a PPR protein defines and stabilizes 5’ and 3’ mRNA termini in chloroplasts. EMBO J 28, 2042–2052 (2009). 10.1038/emboj.2009.121 19424177 PMC2718276

[47] Prikryl J., Rojas M., Schuster G. & Barkan A. Mechanism of RNA stabilization and translational activation by a pentatricopeptide repeat protein. Proc. Natl. Acad. Sci. USA 108, 415–420 (2011). 10.1073/pnas.1012076108 21173259 PMC3017144

[48] Kelley L. A., Mezulis S., Yates C. M., Wass M. N. & Sternberg M. J. The Phyre2 web portal for protein modeling, prediction and analysis. Nat Protoc 10, 845–858 (2015). 10.1038/nprot.2015.053 25950237 PMC5298202

[49] Xiao Y. Low cost delivery of proteins bioencapsulated in plant cells to human non-immune or immune modulatory cells. Biomaterials 80, 68–79 (2016). 10.1016/j.biomaterials.2015.11.051 26706477 PMC4706487

[50] Zhang Y., Ananyev G., Matsuoka A., Dismukes G. C. & Maliga P. Cyanobacterial Photosystem II reaction center design in tobacco chloroplasts increases biomass in low light. Plant Physiology, kiac578 (2022). 10.1093/plphys/kiac578 PMC10069877 36510848

[51] Sinagawa-Garcia S. R., Tungsuchat-Huang T., Paredes-Lopez O. & Maliga P. Next generation synthetic vectors for transformation of the plastid genome of higher plants. Plant Molecular Biology 70, 487–498 (2009). 10.1007/s11103-009-9486-x 19387846

[52] Kang Y. J., Kim D. S., Myung S. C. & Ko K. Expression of a Human Prostatic Acid Phosphatase (PAP)-IgM Fc Fusion Protein in Plants Using In vitro Tissue Subculture. Front Plant Sci 8, 274 (2017). 10.3389/fpls.2017.00274 28293250 PMC5329016

[53] Wang G. Plant expression of cocaine hydrolase-Fc fusion protein for treatment of cocaine abuse. BMC Biotechnol 16, 72 (2016). 10.1186/s12896-016-0302-9 27756365 PMC5069871

[54] Rattanapisit K., Srifa S., Kaewpungsup P., Pavasant P. & Phoolcharoen W. Plant-produced recombinant Osteopontin-Fc fusion protein enhanced osteogenesis. Biotechnology Reports 21, e00312 (2019). 30847284 10.1016/j.btre.2019.e00312 PMC6389792

[55] Kim M. Y. Plant-expressed Fc-fusion protein tetravalent dengue vaccine with inherent adjuvant properties. Plant Biotechnology Journal 16, 1283–1294 (2018). 10.1111/pbi.12869 29223138 PMC5999314

[56] Kannicht C., Kroning M., Solecka-Witulska B. A., Kohla G. & Rosenlocher J. Comparative N-Glycosylation Analysis of the Fc Portions of a Chimeric Human Coagulation Factor VIII and Immunoglobulin G1. Bioengineering (Basel) 4 (2017). 10.3390/bioengineering4020044 PMC5590467 28952523

[57] Gomord V. & Faye L. Posttranslational modification of therapeutic proteins in plants. Curr Opin Plant Biol 7, 171–181 (2004). 10.1016/j.pbi.2004.01.015 15003218

[58] Maliga P. & Tungsuchat-Huang T. Plastid transformation in Nicotiana tabacum and Nicotiana sylvestris by biolistic DNA delivery to leaves. Methods Mol Biol 1132, 147–163 (2014). 10.1007/978-1-62703-995-6_8 24599851

[59] Zoubenko O. V., Allison L. A., Svab Z. & Maliga P. Efficient targeting of foreign genes into the tobacco plastid genome. Nucleic Acids Research 22, 3819–3824 (1994). 7937099 10.1093/nar/22.19.3819 PMC308375

[60] Shinozaki K. & Sugiura M. The nucleotide sequence of the tobacco chloroplast gene for the large subunit of ribulose-1,5-bisphosphate carboxylase/oxygenase. Gene 20, 91–102 (1982). 10.1016/0378-1119(82)90090-7 7160620

[61] Sugita M. & Sugiura M. [Structure of an inverted repeat sequence in chloroplast DNA]. Tanpakushitsu Kakusan Koso 29, 875–888 (1984). 6395189

[62] Lutz K. A., Corneille S., Azhagiri A. K., Svab Z. & Maliga P. A novel approach to plastid transformation utilizes the phiC31 phage integrase. Plant J 37, 906–913 (2004). 10.1111/j.1365-313x.2004.02015.x 14996222

[63] Suzuki J. Y., Sriraman P., Svab Z. & Maliga P. Unique architecture of the plastid ribosomal RNA operon promoter recognized by the multisubunit RNA polymerase in tobacco and other higher plants. Plant Cell 15, 195–205 (2003). 10.1105/tpc.007914 12509531 PMC143491

[64] Lutz K. A., Svab Z. & Maliga P. Construction of marker-free transplastomic tobacco using the Cre-loxP site-specific recombination system. Nat Protoc 1, 900–910 (2006). 10.1038/nprot.2006.118 17406323

[65] Yu Q., Tungsuchat-Huang T., Verma K., Radler M. R. & Maliga P. Independent translation of ORFs in dicistronic operons, synthetic building blocks for polycistronic chloroplast gene expression. Plant J (2020). 10.1111/tpj.14864 32497322

